# Robotic Railway Multi-Sensing and Profiling Unit Based on Artificial Intelligence and Data Fusion

**DOI:** 10.3390/s21206876

**Published:** 2021-10-16

**Authors:** Marius Minea, Cătălin Marian Dumitrescu, Mihai Dima

**Affiliations:** Department Telematics and Electronics for Transports, University Politehnica of Bucharest, 060042 Bucharest, Romania; mihai.dima@stud.trans.upb.ro

**Keywords:** railway automation, multisensory platform, infrastructure failure detection, data fusion, machine learning, statistical data filtering

## Abstract

This article presents the research and results of field tests and simulations regarding an autonomous/robotic railway vehicle, designed to collect multiple information on safety and functional parameters of a surface railway and/or subway section, based on data fusion and machine learning. The maintenance of complex railways, or subway networks with long operating times is a difficult process and intensive resources consuming. The proposed solution delivers human operators in the fault management service and operations from the time-consuming task of railway inspection and measurements, by integrating several sensors and collecting most relevant information on railway, associated automation equipment and infrastructure on a single intelligent platform. The robotic cart integrates autonomy, remote sensing, artificial intelligence, and ability to detect even infrastructural anomalies. Moreover, via a future process of complex statistical filtering of data, it is foreseen that the solution might be configured to offer second-order information about infrastructure changes, such as land sliding, water flooding, or similar modifications. Results of simulations and field tests show the ability of the platform to integrate several fault management operations in a single process, useful in increasing railway capacity and resilience.

## 1. Introduction

The present evolution of intelligent transport systems and policies in this field points towards the reduction of environmental emissions, use of green and/or renewable energies, and increasing the efficiency of processes. The railway transport mode has been given less importance in the past years, due to the rapid growth of road transportation, its flexibility in reaching various destinations, and mobility. However, for large amounts of freight, or numerous passengers, the railway remains one of the most efficient and rapid ways of movement in land transportation. Therefore, the EU policies in the rail transport area are proposing a single European railway area [1]. The document recommends several directions in which the railway transport system should go: (i) interoperability—meaning all high-speed railway automation systems and infrastructures should be compatible, (ii) social harmonization—harmonization of the minimal qualification requirements for workers engaged in interoperable activities, (iii) reducing environmental emissions, especially noise in this case. In this context, it can be noticed that the pressure on the interoperable workers will be greater and the associated necessary knowledge on equipment, infrastructure and operations need to be of a higher level. Therefore, we believe the present solution of automated, preventive maintenance, given by the involvement of an autonomous platform able to collect, process, store and remotely present integrated data and alarming on potentially dangerous modifications of the railway infrastructure could represent a real help in the preventive maintenance activities.

The purpose of this research is to give a solution to the problem of detecting mechanical defects in the subway rail and underground train wheels.

The large-scale development of urban subways raises a major problem, namely the detection of tunnel defects and running tracks, which are becoming particularly important. Due to the complexity of the tunnel environment, it is difficult for traditional tunnel fault detection algorithms to detect such faults quickly and accurately. This article presents an integrated model for the simultaneous detection of defects that can occur in the tunnel but also in the subway rail using a complex battery of LIDAR sensors, ultrasound, video cameras but also a high-performance learning algorithm PCA T2Q for the machine learning module that can detect more tunnel and tread defects quickly and accurately

The novelty of the work consists in developing an autonomous railway cart fitted with several kind of sensors, able to travel along the railway, detect defects by continuous measurements and integrate data via a data fusion process. The same platform can be used with few modifications for the surface railways maintenance activities. A specific method of detecting defects in the railway is proposed, based on an ultrasonic system that analyzes the internal structure of the subway line. The analysis of collected data is based on the evaluation of signal propagation and ultrasonic imaging using decompositions, based on the principal component analysis (PCA) algorithm in the classic version. Supplementary, the analysis is also based on kernel PCA (KPCA). In the first phase, defects are detected, classified, and counted by analyzing their location and geometric features. Then, depending on the maximum difference between the different types of defects and the maximum tolerance of the same type of defects, the generalized characteristics of defects are extracted. Finally, generalization features and training templates are created for the use of a machine learning architecture employing PCA. The purpose is to classify the internal defects of the subway railway. On this basic strong and generalized feature, the constraints are formulated after reducing the size, and the KPCA grouping algorithm is developed to perform data merging for defect detection. The experimental results show that the proposed method can be used to detect internal defects with an acceptable level of accuracy and detection speed.

The remaining of the article is organized as following: Section 2 presents related work in the domain, based on a literature study, Section 3 is dedicated to the design of the proposed solution for mobile autonomous data collection, Section 4 is for presenting the test-bed setup and results of experiments, Section 5 Discussion, and finally conclusions.

## 2. Related Work

### 2.1. Context

Due to its high degree of safety, the railway is an effective mode of transportation for both passengers and freight. With the inclusion of high-speed trains with dedicated and surveilled infrastructures, land transportation became now competitive with air transportation, mostly for medium distances. In Europe, Germany began operation of high-speed concept in railway transportation with ICE trains in 1991 and in 1994 the UK was linked to the European continent via the Eurostar service, connecting Paris to London through the Channel Tunnel. The document [2] states that “Due to France’s early adoption of high-speed rail and its central position between the Iberian Peninsula, the British Isles and Central Europe, most other high-speed rail lines in Europe have been built to the French standards for speeds, voltage and signaling, with the exception of Germany, which built to existing German railway standards.” The trend continues nowadays to increase the operational speeds of trains, no matter if it is about surface railway or underground, so there is more pressure on the quality of infrastructure, maintenance, and operation. Therefore, we consider that automatizing the process of preventive maintenance becomes a must for the railway industry.

The subway transportation is another form of railway transport, designated mostly for urban areas. It also represents the best alternative to automotive, individual transportation in large urban environments. However, there are numerous cases in which railway, or subway networks face extensive wear and require large amount of capital expenditure to maintain sufficient performance levels. Furthermore, based on these aspects, it was considered that the development of an integrated solution to make as efficient as possible the fault maintenance operations is necessary. In these conditions, the objective of the present research presented in this article was to develop an autonomous, intelligent platform for railway and/or subway stations and tunnels measurement and profiling, considering infrastructural, electrical, and mechanical components.

### 2.2. Literature Survey

The scientific literature is relatively rich in the field of automated measurement and non-conventional solutions for a rapid assessment of the status of different functional components of a railway, or underground network. The modern signaling systems, interlocking and concentrated control dispatching solutions now use widely standardized solutions employing fail-safe and majority (triple) redundancy principles in railway automation. These involve numerous elements with high functional responsibility, zero-tolerance to critical faults that lead to catastrophic events in railway. Therefore, the responsibility in fault maintenance systems is very high, wherefrom the intense research in this direction to achieve new solutions and procedures for fast and efficient measurements of correct state of operation. In addition, the railway mechanical infrastructure itself, represents a collection of constructions, mechanical elements, ancillary equipment always exposed to intense wear and stress, that need continuous monitoring for early detection of faulty elements. Until present, there are few developments in the field of autonomous railway vehicles designed to detect and mark defects, and the subway lines make no exception. Track alignment and other geometric measurement methods usability is analyzed in [3], where the authors conclude that “The selection of the optimal method of measuring the geometry of the track system depends on the technical and economic conditions of the contractor and the conditions prevailing in the field.” They also say that “… automatic systems such as measuring systems using measuring cars will be the optimal choice.” Pengyu Pan et al. [4] present a solution for measuring the impedance of electrical traction network at high-speed trains. They propose a method to determine the equivalent impedances of traction network and the 4QC of electric train in the stationary frame for stability analysis, also determining the stability and mechanical oscillation issues specific to high speeds. Complex magnetic permeability measurements are used for calculating rail internal impedance via finite elements method presented in [5] by Alberto Dolara and Sonia Leva. They considered the normal magnetization curve and complex magnetic permeability and included that data into the proposed finite elements method models. The authors also present an electric model for the dynamic behavior of the supercapacitors for this methodology of measurement. Passing on to the rolling contact analysis for railway systems, in [6], the author investigates the defects (cracks) with the help of a robotic inspection system. A novel technology, involving mobile mapping systems, is presented by Daniel Lamas et al., being applied on a 90 km long railway sector for the acquisition of data regarding the classification of rails, masts, wiring, droppers, traffic lights, and signals. The methodology consists of a pre-processing phase, in which each point cloud is sectioned and voxelised (a process in which it is produced any of the discrete elements comprising a three-dimensional entity); then a segmentation process is performed, followed by a merging process. The methodology presented automatically extracts relevant assets of the railway infrastructure, such as rails, wiring and signs, traffic lights, and marks, from 3D point cloud data [7]. Zhang Yi et al. present in their article [8] an adapted method for grounding impedance measurement of high-speed trains integrated grounding system, with a four grounding terminals impedance tester, with a compensation methodology for more accurate results. A solution for exterior railway infrastructure inspections is presented here, where the authors employ a combination of an inertial system with a GNSS receiver for inspecting high-speed railway lines. According to the authors and their experiments, the presented methodology and instrumentation can produce a measurement of the track parameters with an accuracy better than 0.2 mm at a detection speed of around 3 km/h. The authors state that “compared with the traditional Kalman filter method, the proposed design improved the measurement accuracy and met the requirements for the detection of geometric parameters of high-speed railway tracks.”

A visual measurement system for track gauge evaluation was developed, experimented, and presented in [9], involving setting of some measurement points, detection of those points and measurement of distance between them. The experiments were carried on Vilnius railway station yard. The authors write that the proposed method’s advantages are its easy integration, low cost, and energy efficiency. In addition, in this field of measurement activities, Chen, et al. [10] describe their work, employing the integration of an inertial navigation system with geodetic surveying apparatus to set up a modular train gauge measuring trolley system. Kampczyk [11] presents the analysis and evaluation of the turnout geometry conditions, also describing the causes of turnout deformations. Researchers such as Wootae Jeong and Dahae Jeong [12] present a method for accurately measuring the roughness of wheels and rails, considered the main cause of producing the noise during trains operation. They propose enhancing the chord offset synchronization algorithm applied to the existing ARCer for high measurement precision with only two displacement sensors. There have been also proposed alternative solutions such as the one of chord offset synchronizing, which assumes that the rail surface is a sinusoidal wave consisting of various wavelengths and uses multiple sensors so that each one compensates for the measured values of the others. This procedure has been used to prepare for the structural shortcomings of mobile measuring systems equipped with displacement sensors [13,14]. Visual sensing, such as two-dimensional image recognition, is also employed in the measurement and/or detection of the abnormal fastener in the rail-track inspection system. The authors of [15] propose a multi-source visual data detection method, and an accurate and robust fastener location and nut or bolt segmentation algorithm. They write that “By combining two-dimensional intensity information and three-dimensional depth information generated by the projection of line structural light, the locating of nut or bolt position and accurate perception of height information can be realized in the dynamic running environment of railway.” In addition, in a similar work, Liu [16] describes a method for foreign objects detection, based on a deep trust network for railway environment protection and trains safety. The author of [17] uses a visual methodology for fastener condition detection, based on the model of Siamese deep network, where at the input is a couple of images. The features of the fastener are extracted by determining the similarity of a pair of images. A method for the alignment and centring of the railway track is described in [18], where a combination of a global navigation satellite system, an inertial measurement unit and a laser scanner is used for increasing the precision to an acceptable level. The combination of the above-described methods gave the best result for the track on which the measurement vehicle had moved in the practical experiments, mapping almost 100% of the track. Similarly, Elberink and Khoshelham [19] published their research results on the automatic extraction of center line of railway tracks from laser scanner data using data-driven and model-driven approaches. Aerial observation for detecting intrusive objects in the railway movement space is presented in [20], by Neubert, M. and others, such as Zhu, L. [21], who proposes the use of an airborne laser and mobile laser scanning for modelling the railway environment. Usually, by employing data fusion from visual and laser imaging, a complete railway environment can be aggregated and further analysed employing various techniques, to detect foreign objects on the railway space, to evaluate the state of health of different infrastructure elements, etc. Corrections, in this case, might prove necessary to employ, such as described by different other works [22,23,24].

GNSS technologies are good for use in outdoors experiments, measurements, and recordings. However, as presented before, such technologies cannot be employed for railways in tunnels, or for subway lines, where the presence of deep concrete walls prevents the low-powered signals of GNSS to be received. Here is a novelty of our proposed solution, where the robotic cart determines its position via odometers and has the ability to integrate measured data with position. For indoor environments, different technologies are to be adopted for positioning, measurement of gauges and geolocation. One of these is, of course, laser scanning. As presented in [25] by Qian Wang, et al., a mobile laser scanning system (MLSS) was used for the inspection of subway tunnels, and the key technology of the positioning and orientation system (POS) was investigated. The authors obtained an “accuracy of the 3D coordinates of the point clouds of 8 mm, and the experiment also showed that it takes less than 4 h to complete all the inspection work for a 5–6 km long tunnel.” The missing of the very practical GNSS technology for accurate positioning in tunnels or radio-obscured spaces must be replaced by laser scanning, ultrasonic scanning, methods that must be also completed with map matching (MM), intermittent responders, coordinates of control points, and so forth [26].

Things become even more complicated when specific measurements are needed to be performed to determine curvature, or elements situated in a curved section of a railway, or a tunnel railway. Arkadiusz Kampczyk [27] is proposing an innovative measuring device called the magnetic-measuring square (MMS).

The researcher describes a method for lacing/string lining and the measurement of the perpendicularity of rail joints. He also employs an MMS device for measuring versines and differences recorded in the lengths of rails, especially in curves. A laser beam is used for this purpose with a target cross, a camera, and a surveying disk for measurement.

For pro-active maintenance purposes, Pacifique Turabimana and Celestin Nkundineza propose in their article [28] the testing of a new measurement tool that employs an inductive displacement sensor. The proposed system is said to be working in both static and dynamic state of the railway vehicle and being able to save the historical records of the wheel flange thickness for further analysis. In related work, employing different measurement technologies and platforms, such as cameras, lasers, ultrasonic transducers, is presented in [29] and [30]. In a similar work, using a lidar to for 3D modelling of train rails is described in [31]. In their research [32], Li Q., et al. analyse the accurate measurement of the railway track geometry possibilities and show a solution based on the integration of an inertial navigation system (INS) with a geodetic surveying equipment. They also designed a modular TGMT (TGMT—trainguard mass transit—allows for a close distance between trains and allows the use of driverless trains) system based on aided INS, with the ability to be configured according to different surveying tasks, including precise adjustment of slab track, providing tamping measurements, measuring track deformation and irregularities, and determination of the track axis. The authors also declare that the method proposed by them can improve the surveying capacity and efficiency at least 20 times, compared to the traditional methods. Outside, the tracks’ irregularities may be measured with the help of GPS receivers for positioning and alignment, but in subway tunnels this procedure is not available. Q. Chen and others employ a laser-aided INS/odometer integrated system to determine the subway track irregularity and the geo-reference absolute position relative to the geodetic control network is determined by a laser scanner, to estimate the INS drift. In a similar work, Jiang Q. et al. present a new filtering algorithm for railway track surveying using also landmarks and inertial measurement units, complemented by odometers [33], and applications using inertial sensors and odometers are analysed in [34].

Regarding the electrified line and its associated equipment for power traction, Morris J., et al. [35] present a research on modelling the short neutral section for AC line electrification—a source of frequent faults of the power supply network. In addition, considering the automated measurement techniques, Chen L. and his co-researchers use a methodology to collect information regarding the overhead contact system component, employing data analysis on point clouds imported from a 2D mobile lidar. They also designed an iterative point partitioning algorithm and a module named as the spatial fusion network [36].

Coming back to mobile solutions for sensing remote parameters of railway equipment, it is most usual to employ specific wagons, equipped with diverse measuring instrumentation, such as in [37,38,39,40,41,42,43,44]. While a dedicated wagon can accommodate a larger quantity of equipment and sensors, its availability, mobility, and flexibility of manoeuvring are less adequate as the one of a smaller, fully autonomous devices, such as railway carts. Especially for subway purposes, where space and gauges are in general smaller than those in the open space railway systems, the utility of the cart is much bigger. Therefore, one aspect of this article is that it focuses on the relatively novel process of employing an integrated approach for solving as much as possible automated measurements with a single, compact autonomous cart. Using a dedicated solution with several sensors, data fusion and analysis of recorded data based on machine learning techniques can ease the work of human operators and automatically collect relevant information of fault management, during off-service hours in subway transportation. The following represent aspects of novelty in the present research:

- Autonomy of the method: the robotic cart can travel alone, collect position-referenced information regarding tracks gauges, deviations from standards, presence of foreign objects on the rails, imaging and external gauge profiling;
- Collected data can be either transmitted via Wi-Fi to dedicated access points, or locally stored for off-line analysis;
- Integrates various machine learning techniques for data fusion;
- Has the possibility to fine tune a wide variety of measurements parameters and speed increments;
- Is designed especially for subway lines with no GPS positioning signals, but can be adapted to work in external environments, using additional GPS information for position of a location;
- Reduces stress of maintenance personnel by overtaking some tasks in usual maintenance activities during subway off-service periods;
- The following are comparisons with other previously published works related to the railway inspection approaches:
- Rowshandel, H. [6] proposes in his doctorate thesis a robotic inspection system for discovering fatigue cracks in the rolling system of a surface railway. The system employs an alternating current field measurement (ACFM) sensor combined with a rule-based expert system. The solution is dedicated for collecting a single type of data, only detecting cracks, and not inspecting railway gauges, for example;
- Only 3% of the maintenance operations in railway tunnels (including subway) are recently subject of robotic activities [45]—therefore, the benefits of such a solution;
- Robotic autonomous systems have been employed mostly for cleaning purposes [46], rolling stock fluid servicing [47];
- Killian, K [48] proposes a vision system based on wayside sensors for inspecting integrity of train wheels and rails (no mobility involved here for the automatic measuring system);
- Railway catenary and power line automatic inspection are proposed via a system with laser beams and imagistic analysis simultaneously for four wires [49]—the solution is intended to be mounted on the engine of a train and is operational for speeds up to 90 km/h. No other parameters are envisaged to be measured;
- Railway robotic inspection in tracks maintenance operations: machine vision and classification algorithms are used to detect and/or localize cracks in the rolling surface of tracks. It is employed a laser scanner mounted on a car that uses a random Forest classification learning algorithm [50] and for tracks geometry [51]. No other parameters are envisaged to be measured;
- Ground penetrating radar is also used for some robotic inspections in tunnels [52].

Usually, these systems for automatic measurement and data collection are designed for fewer dedicated operations and/or specific activities. Our proposed solution can make use of different sensors to determine gauge parameters, detection of foreign objects on tracks, smoke and gas, and as future development, the cart will be fitted with a system to measure tracks impedance (for track circuits tuning).

Another novelty that our solution brings is the integration of sensors in a complex system, and for prediction we use a machine learning algorithm based on PCA T2Q.

## 3. Design of the Proposed Solution for Mobile Autonomous Data Collection

### 3.1. Rail Defects

Depending on each specific railway administration regulations, there are several types of infrastructure (railway) faults, or defects that involve significant safety concerns. The safety compliance is even more drastic when the maximum allowed speed of the train is increasing; therefore, high-speed lines have a distinct corridor, points for these lines have a longer tangent and double mechanical locking, and specific requirements are set for the catenary and vibrations. From this point of view, high-speed lines need an intensive process of maintenance, starting with the visual inspection and ending with dedicated measurements for mechanical integrity, locking of points and deformations. Even detection of foreign objects that might fall between the lines, or between the point switches arms is important. On the other side, for subway lines the same restrictions apply, with the specification that early visual detection of a foreign object on the rails might be delayed due to low intensity of light in tunnels. In addition, subway lines use in general a third rail for power supplying, and this one also needs constant monitoring for defect detection.

Usual infrastructure defects that might occur in the normal railway exploitation (both for surface and underground lines) include:

- Rail fracturing (might be early detected employing ultrasonic and/or video solutions). In case of complete fracture, the track circuits might be also able to detect this type of defect via electric control and interdict the entrance of a train on the specific section via the covering signal;
- Intensive burring of insulating joints from neighboring rail coupons might induce short-circuits between adjacent track circuits, producing delaying in trains operations;
- Mechanical deformations of gauges (between parallel rails, or between elements of a switching point, or a crossing)—this can be caused by exceeding the allowed weight per axle, mud under the rails, or other types of phenomena. It can also be caused by falling of heavy objects on the respective parts, especially from freight trains. In intense and prolonged warm summers, the temperature on the rails level might exceed 50 °C, causing mechanical deformations due to dilatation. This is a very dangerous defect, that must be early detected. Therefore, in very hot summers railway administrations impose speed and weight restrictions;
- Intensive erosion, rust, or wear of the rail rolling surface. This type of defect might be considered from two points of view: firstly, if the line is intensively eroded, the electrical contact between the rail and the train wheel is imperfect and might cause malfunctioning of the track circuit, which might also trigger false response on the railway signaling, with possible catastrophic effects leading to trains collisions. It is very important to use safe track circuits from this point of view (based on high voltage pulses) on such lines, or to perform regular traffic to reduce erosion by mechanical friction. On the other hand, if the rails exhibit intensively wear, the surface becomes irregular, or with undulations, causing vibrations on the rolling stock or possible loss of electrical contact at high speeds. Therefore, also these types of mechanical deformations should be detected and resolved in due time.

For determining such specific mechanical and geometrical defects, usually regular observations are performed by railway personnel, traveling by foot distances between railway stations and noting where observed such anomalies. In addition, semi-automated measurements of higher precision are performed with dedicated cars (wagons) that are periodically traveled along the rails. These methods are more difficult to perform to underground railways, however, due to lower visibility conditions and very short times of operating breaks, especially during night.

From these points of view, we consider that the development of an automated platform able to displace, collect and transmit relevant information regarding the infrastructure integrity is highly necessary.

### 3.2. Rail Diagnostics Techniques. Description of Hardware

**Figure 1:**
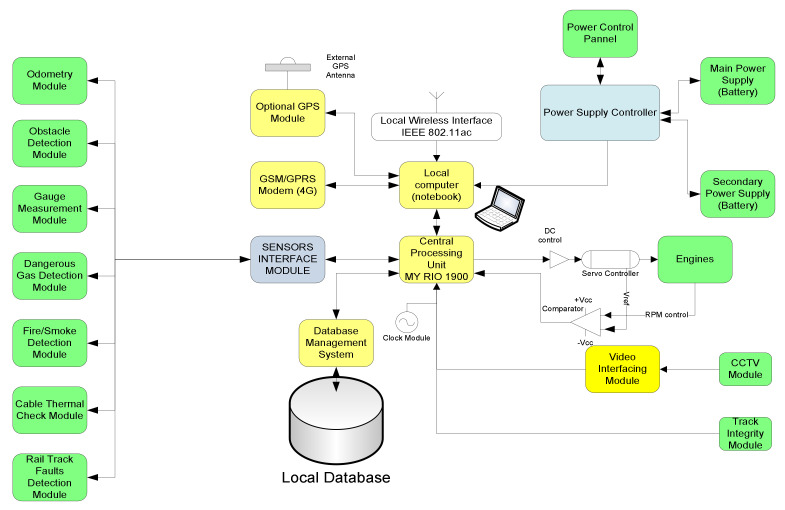
General functional blocks diagram of the autonomous cart.

The proposed solution for mobile autonomous data collection for railway applications is composed of a set of sensors mounted on a self-driving cart, enabled with self-locating techniques (including indoor positioning, powered by a combination of odometry and INS combination in the first phase). The first version was initially conceived mainly for subway applications, including analysis of external gauges limits, testing of on-board/ground communications and EM beacons, obstacle and/or foreign objects detection, and early fire discovery and warning. The block diagram of the first version is depicted in Figure 1.

In the first version, the hardware equipment encompasses several functional components, such as:

- Central processing unit (CPU) based on myRIO—holds all the controls and commands for the mobile platform, programmable via a notebook;
- Odometry module (OM)—responsible with counting pulses from the wheels, measuring traveled distance, speed and updating the information about position of the automated platform to the CPU. For computing traveled distance and speed, a NPN Hall-Effect sensor has been employed. The following formula is employed for determining velocity:

(1) $$V\left[\frac{m}{s}\right]=n\cdot \frac{2\pi R}{60}$$

where *n*—count number of pulses transmitted from the Hall sensor, *R*—radius of the flange that ensures counting pulses.

**Figure 2:**
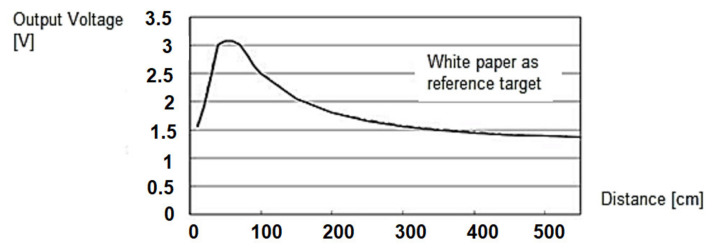
Sensitivity over distance for the obstacle detection sensor (reference target: white paper).

**Figure 3:**
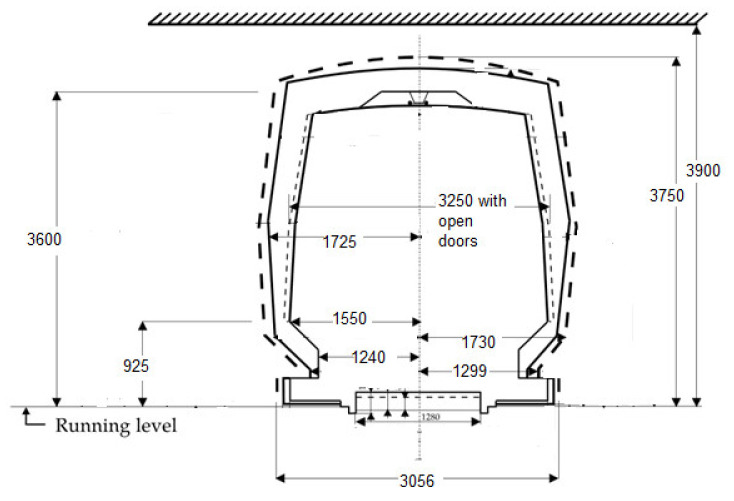
Shape and main dimensions for the Bucharest Underground Gauge (transversal section, a typical section of a tunnel, semi-rectangular section. There are also ovoidal and circular sections).

- Obstacle detection module (ODM)—it is composed of an infrared sensor and an ultrasonic sensor combination mounted in the front of the mobile platform for rapidly detecting and/or identifying obstacles on tracks. The detection of obstacles that are present in the front of the platform is performed through an IR sensor type Sharp GP2YOA710KOF and an ultrasonic sensor XL Maxbotix EZ0. Sharp GP2YOA710KOF is an integrated distance sensor for front obstacles, that ensures IR LED detection at λ = 850 nm. Operating distance is comprised between 100 and 550 cm. Its sensitivity diagram is presented in Figure 2. A 90% reflection coefficient was considered for the white paper set as a reference target. The dimensions, quality, and ease of use of the ultrasonic sensor XL Maxbotix EZ0 also allow high accuracy readings from 0 to 765 cm with a resolution of 1 cm. The sensor can be supplied with a voltage between 3.3 and 5 V D.C.

- Gauge assessment module (GAM)—composed of ultrasonic and laser sensors to check the dimensions of the external gauge of tunnels, detect and locate eventual obstacles, or protruding objects. For a much more precise gauge check, an RPLIDAR A1M8-360 laser kit with a maximum reading frequency of 10 Hz and a detection distance of approximately 6 m was also introduced. The Bucharest underground free pass gauge shape is presented in Figure 3.
- Fire detection module (FDM)—composed of dedicated IR sensor and cooperating with GAM for fire detection, heated cabling location and alarming.

For the software design the LabVIEW program has been employed, whose source code was executed by the NI myRIO data acquisition module, using a combination of modular hardware and software to transform the personal computer (tablet, laptop, smartphone) into a user-defined remote-control system.

Below are some images with the real equipment tested in subway tunnels.

**Figure 4:**
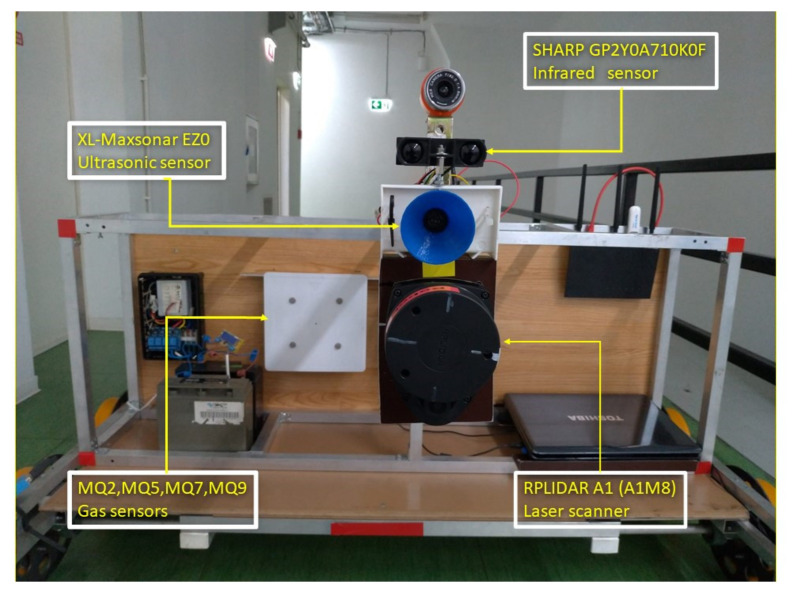
Frontal view of the equipment.

In Figure 4 in the central part, the white box represents the gas/smoke detection module, in the upper right corner is the Wi-Fi router, the central unit is in the center of the image.

**Figure 5:**
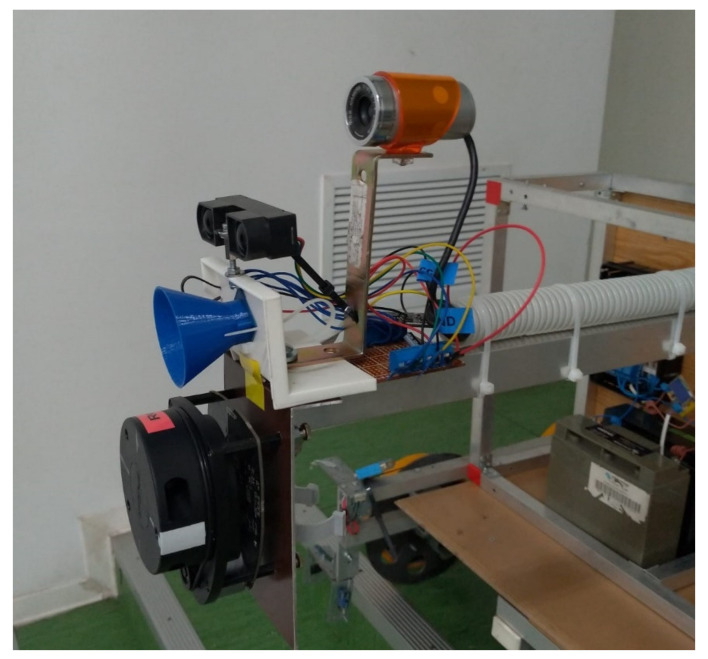
Detail with lateral view: web camera above, obstacle and fire/smoke detection modules.

**Figure 6:**
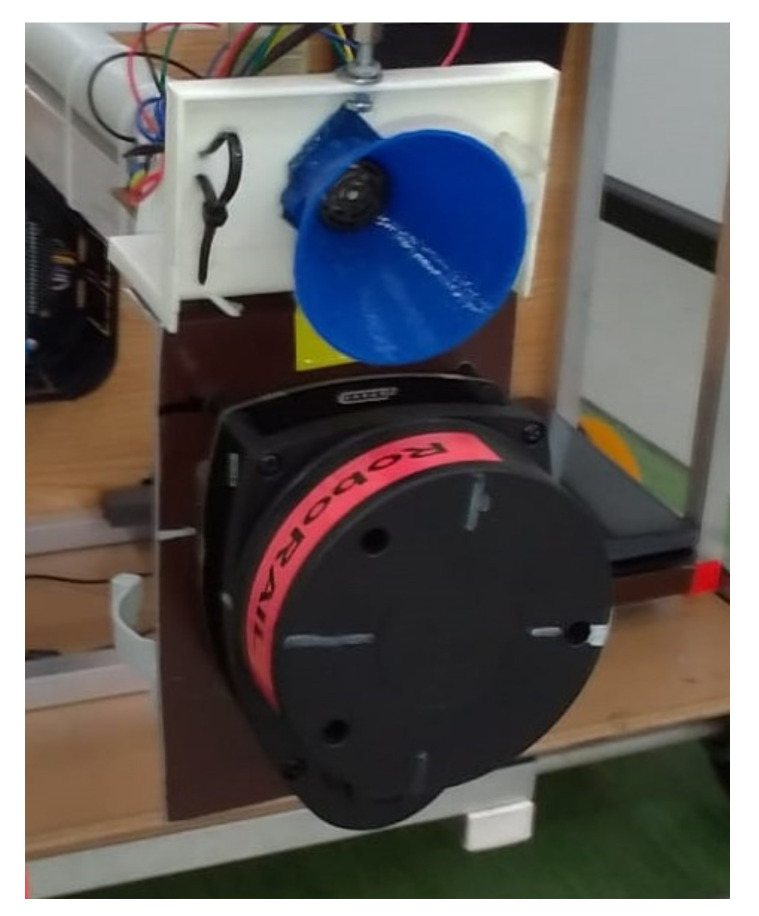
Detail for gauge assessment module with LIDAR scanner and obstacle detection module (ultrasonic).

Figure 4, Figure 5 and Figure 6 present some physical details of the experimental robotic cart. The movement of the cart is ensured by the motors mounted in each axle of wheels (not visible in the presented images). It is possible to regulate the displacement speed and to set the sampling speed of the set of sensors.

## 4. Test Bed Setup and Results

### 4.1. Introduction

For most tasks assigned to a mobile system for detecting path defects, the use of a single type of sensor may not give satisfactory results. For example, in navigation, some objects in the environment can only be detected by IR sensors, lasers, and others only by ultrasonic sensors, and only few by all types of sensors. The problem is, therefore, to find a method that effectively combines information from a multitude of sensors with different categories and characteristics. The most common term in literature for this process is “sensor fusion”. However, in the context of mobile systems, the merging of data must cope with the following challenges:

- Merging sensor measurements of different categories;
- Merging measurements from different positions and angles;
- Merging measurements taken at different time intervals.

**Figure 7:**
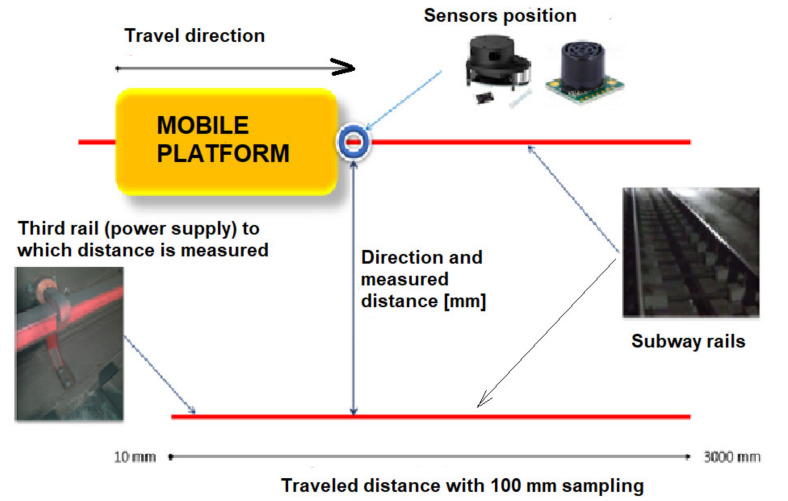
Test bed setup, placement of sensors and elements measured.

For the experimental tests, the robotic platform was mounted on the Bucharest subway near Straulesti station in the tunnel, with the purpose of determining by measurements the gauge to the third rail (used for power supplying the trains by a patina). The test bed setup is presented in Figure 7 below.

**Figure 8:**
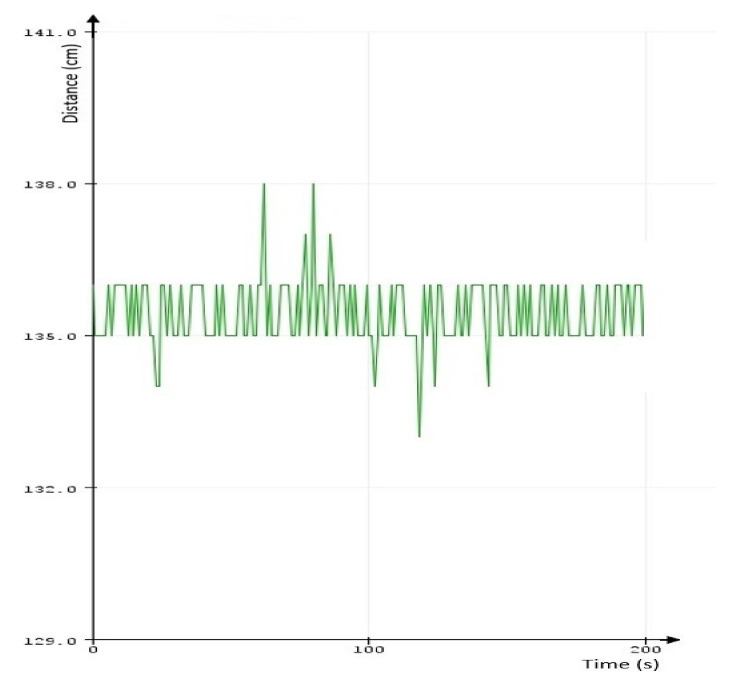
Direct data sampling from XL Maxsonar EX sensor measurements.

Using the measurement and data acquisition system presented in Figure 1, the results presented in Table 1 were obtained. A sample of direct data exit from the 3 XL-Maxsonar EZ sensor, one from the set of sensors installed on that platform, is presented in Figure 8.

**Figure 9:**
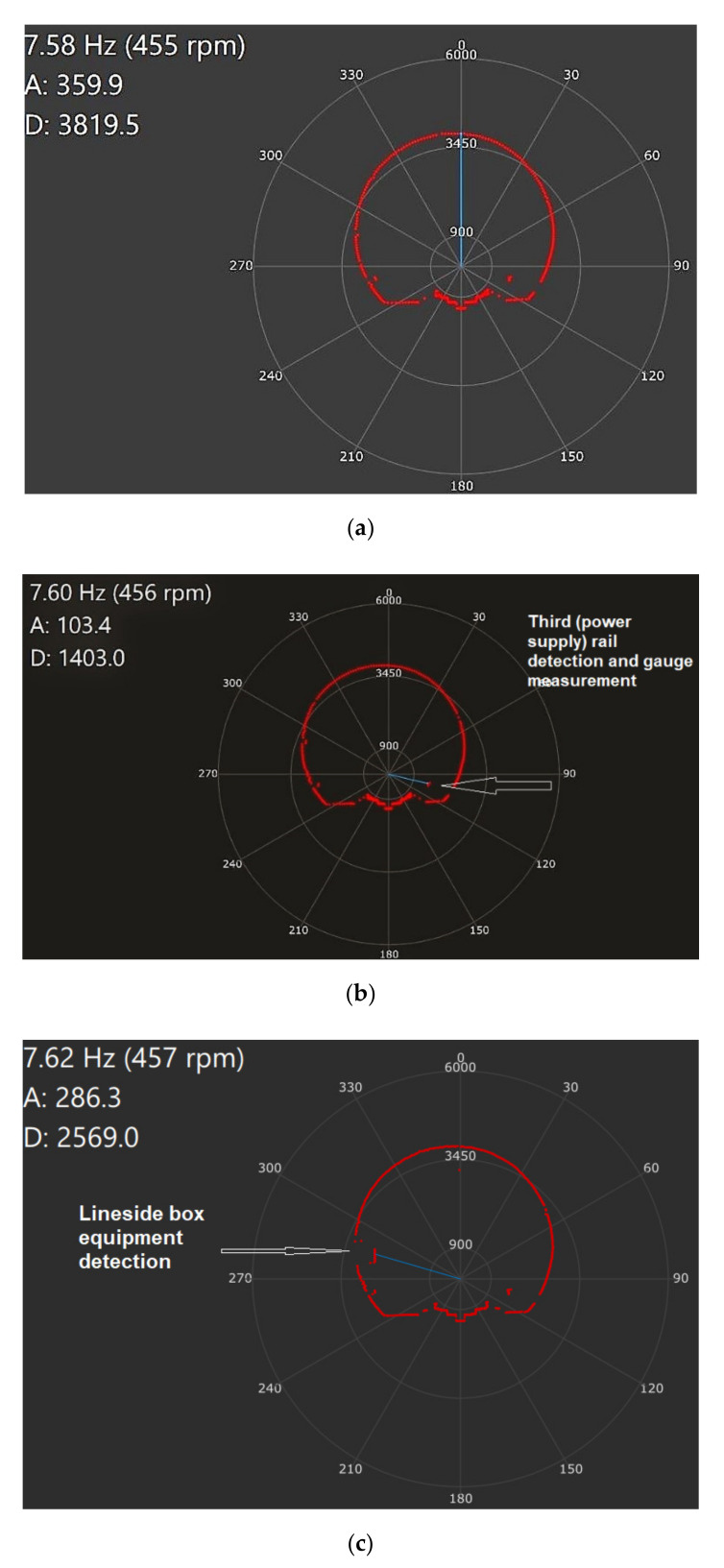
Gauge measurement with LIDAR, (**a**) tunnel gauge profiling, (**b**) third rail (used in subway for power supplying), (**c**) lineside box equipment (LDE) gauge measurement (LDE is mounted on the tunnel wall, round-shaped subway tunnel profile between stations).

Figure 9 represents the shape of the lateral gauge captured live with the Lidar. Details of different elements monitored are described in the figure: overall transversal gauge Figure 9a third rail used for DC power supplying the trains Figure 9b, and Figure 9c the lineside equipment box mounted in a certain longitudinal position on the tunnel wall. The complete set of data collected in the experimental test is presented in Table 1.

As it can be noticed in Table 1, the results for longer railway paths involve collection of large quantities of data, exhibiting a relatively large variation, fact that requires reducing the size without losing the relevant features, in order to remain usable in the architecture of supervised machine learning.

**Figure 10:**
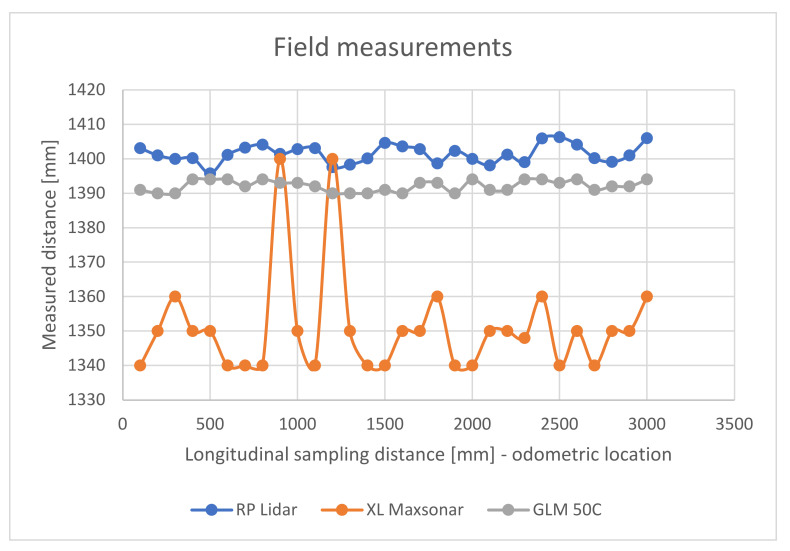
Diagram showing the variation of distance measurements between three sensors.

The resulting variation of the distance measured to the third rail has been transposed in the diagram below (Figure 10). It can be observed that the XL Maxsonar sensor exhibits the largest variations in precision, while the RP Lidar A1 sensor is more constant in precision. The GLM 50C laser measurement instrument was used as reference for additional comparation. It is obvious that in the data fusion problem there are some elements that are to be considered, amongst which is the precision, delaying, position of measurement and timing. All these factors should be harmonized by the data fusion process. In the case of a mobile platform, the mechanical structure where the sensors are installed, its stability to vibrations and overall inclination are also additional factors that should be taken into consideration or compensated. Vibrations might be reduced in a practical application by installing the most sensitive sensors on a gyroscopic stabilized platform.

For data fusion and detection of defects (anomalies) it is proposed the development of a machine learning algorithm based on principal component analysis (PCA).

PCA is an algorithm for reducing the linear dimensionality and the detection of anomalies. The kernel PCA (KPCA) is the nonlinear representation of the PCA, which can be successfully applied to complex spatial structures, and the kernel function is associated with the nonlinear transformations applied on the common PCA algorithm.

Application of KPCA requires high computing power and significant memory, especially if applied to large drive data sets. To apply the KPCA for large sets of training data, we propose using a reduced version of KPCA in the process of structural anomalies detection inside subway tunnels.

### 4.2. Development of the Machine Learning Model with PCA T2Q

The suggested methodology will be employed for reducing the dimensions of the collected data obtained with the system shown in Figure 1, still maintaining its relevant features. Based on the proposed reduced KPCA algorithm, a machine learning system was developed to combine the training and testing the process of dimensional data reduction, the supervised learning, and the possibility to choose the core function. For detecting defects (anomalies), the T^2^ model and the square predictive error Q (Q statistics) are employed. The combination of T^2^Q results is then used as a defect detection value (index). The T^2^-statistic measures the variation in each sample and indicates the distance of each sample from the center of the PCA model. The Q-statistic indicates how well each sample conforms to the PCA model by measuring the distance that a data point falls from the PCA model.

**Figure 11:**
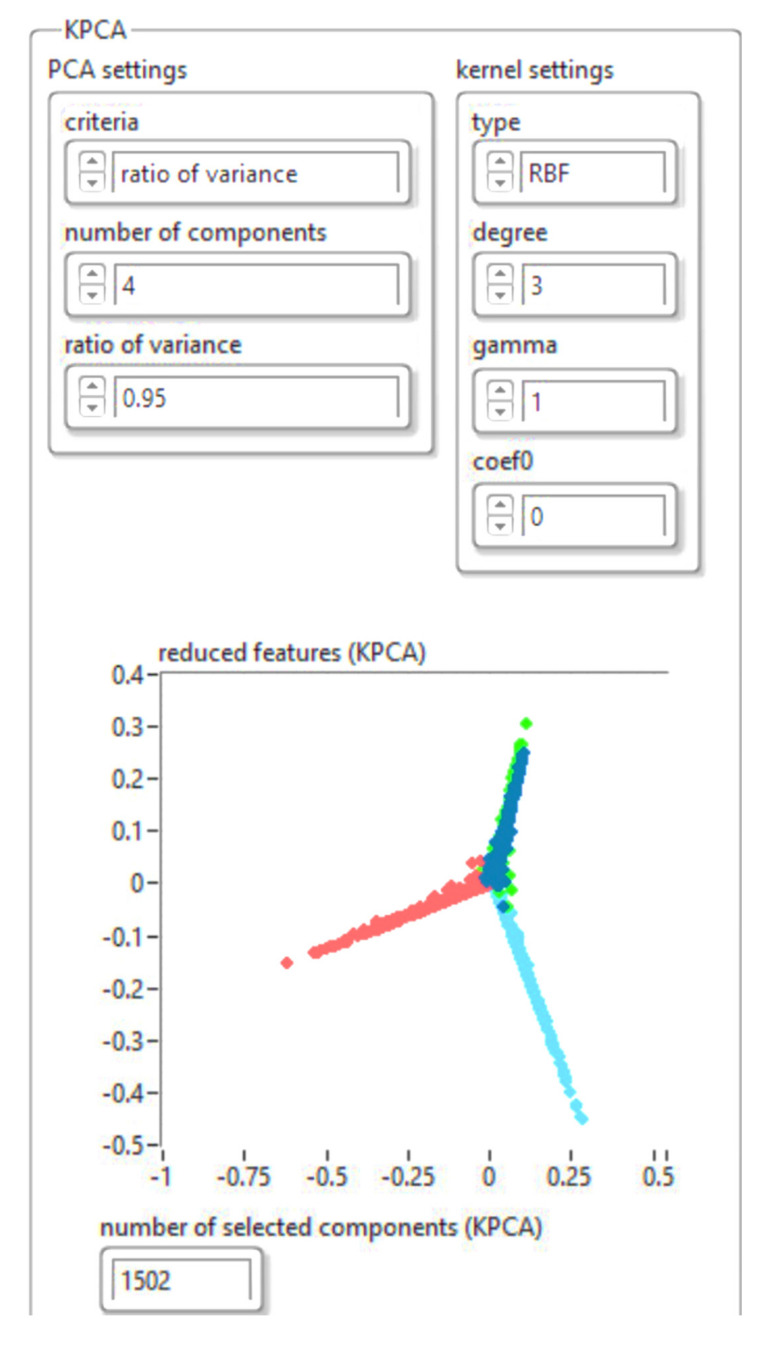
Implementation of the KPCA algorithm for feature manipulation.

In the first stage, the dimensions are reduced using the KPCA algorithm. To achieve this, the algorithm was implemented in LabVIEW programming language. Figure 11 presents the feature manipulation algorithm.

**Figure 12:**
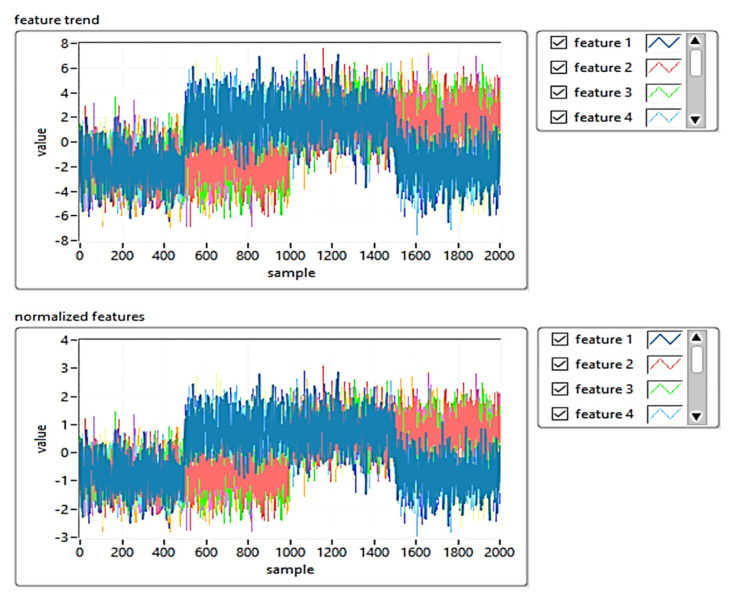
The results obtained for reducing the database size using the KPCA algorithm.

The results obtained for reducing the database size using the KPCA algorithm are shown in Figure 12. The drive file was saved in “json” format.

To construct the PCA T^2^Q model, the input matrix was divided into a training set (for developing the model), and a test set, in an odd-even manner. The input matrix was mean-centered and scaled to a unit variance. This is necessary for the PCA model development. PCA functions in LabVIEW and MATLAB were used to calculate the principal components, the eigenvalues, and the amount of variance explained by each PCA component.

In the second stage, the machine learning architecture was implemented based on the PCA T^2^Q algorithm, the training, and defect detection applications.

**Figure 13:**
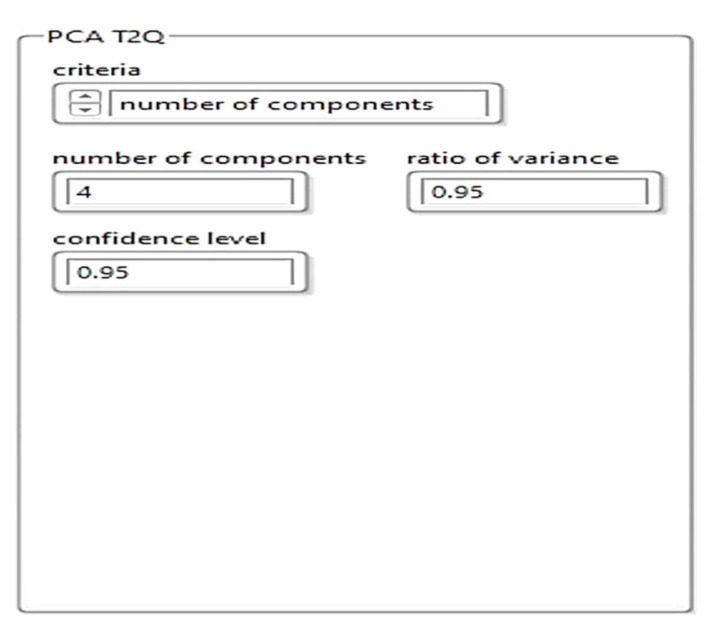
Software implementation of the PCA T^2^Q training algorithm.

**Figure 14:**
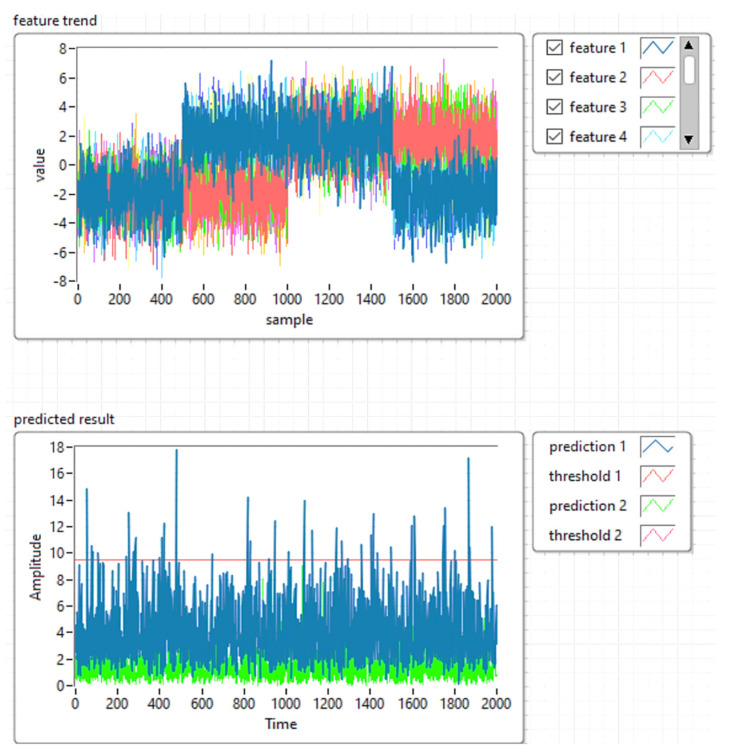
Results of the training templates using PCA T^2^Q.

Figure 13 shows the implementation of the PCA T^2^Q training algorithm, and Figure 14 shows the results of the training templates.

### 4.3. Simulation and Experimental Verification of the Model

In this experimental research a machine learning algorithm is proposed with the principal component analysis (PCA), used to develop a predictive estimator based on the load state model.

Detection of structural damages using principal components analysis and failure indices is based on the development of an initial basic model for undamaged structure. This one is constructed by applying principal components analysis to data collected via several experiments, and after the current structure (damaged or not) is subjected to the same experiments, and the collected data are projected into the main component analysis models. Two of these projections and four damage indices (T^2^ statistic, Q statistic, combined index and I2 index) of each actuation phase are used to determine the presence of an anomaly and to distinguish between them. These indices are calculated based on the analysis of the residual data matrix to represent the variability of the projected data in the residual subspace and the new space of the main components.

**Figure 15:**
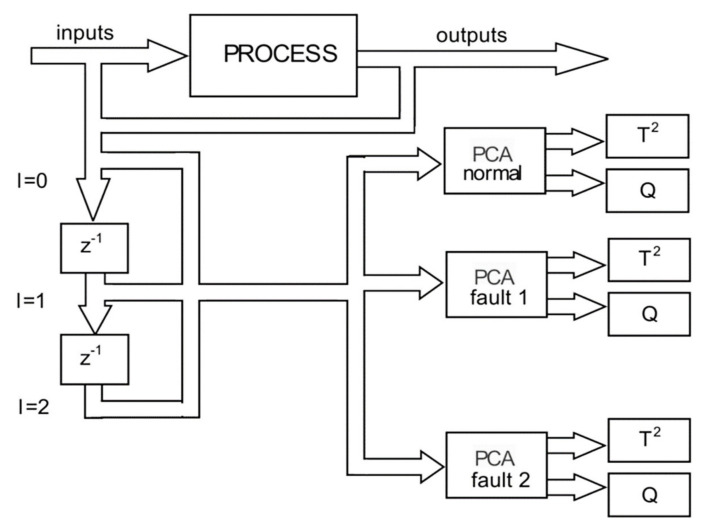
The software architecture for the detection of defects and anomalies.

Figure 15 presents the software architecture for structural anomaly detection. The actual data are compared to a reference model, which is formulated as covariance matrices and mean values of the measured variables.

### 4.4. Case Study

- Rail defect detection techniques based on ultrasonic waves analysis and machine learning. Experimental research on the behavior of ultrasonic sensors in the detection process.

In order to extend the measurement capabilities for the detection of internal and/or mechanical deformations and cracks in the rails, the research was also pointed to test the efficiency of using higher frequency ultrasounding in anomalies defectoscopy. This involves some challenges, though:

- The placement of the sensor very close to the target for minimizing air attenuation;
- Placing the sensory assembly on a stabilized platform, or with good mechanical attenuation to vibrations is required;
- A deeper experimental analysis of the performances that higher-frequency ultransonic scanner has on different materials, in different conditions is required.

The performances of mobile platforms depend not only on the purpose and objectives they have to fulfill, but also on the space in which they carry out their activity. Choosing the right sensory system requires a serious analysis of the space in which the mobile platform will operate and its particularities, i.e., propagation medium attenuation, obstacles (objects), which can be either mobile or fixed. Following the measurements performed with different ultrasonic sensors on multiple types of obstacles, the dependencies between them and the different detection possibilities must be established.

- Measurement errors

For the measurements made with ultrasonic sensors, as for any other measurement technique, a certain amount of errors affects in the results. Performing measurement produces errors that have the same amplitudes when the process measurement is performed under identical conditions, or errors that have variable amplitudes, their variation depending on certain laws. Errors resulting from the measurement processes may be classified into:

- Gross errors that result from misreading or inattention and must be eliminated;
- Systematic errors that occur due to some constructive characteristics of equipment, or may be caused by external factors (temperature, pressure, humidity, noise etc);
- Random errors that occur as a result of the diversity of processes and phenomena as well as a the interactions of the experiment with other processes and phenomena that take place simultaneously.

In the process of analyzing the data from the measurements performed with the ultrasonic sensors it is necessary to calculate the absolute error, the relative error and to determine the maximum admissible error. The absolute error is given by the relation:

(2) $$\Delta x=\left|x_{m}-x_{r}\right|$$

where $x_{m}$ is the value obtained by measurement and $x_{r}$ is the real value.

The relative error is given by the relation:

(3) $$\varepsilon =\frac{\Delta x}{x_{r}}\times 100$$

where ∆*x* is the absolute error and $x_{r}$ is the real value. The relative error is expressed as a percentage. The maximum permissible error is determined by choosing the maximum value of the absolute error.

(4) $$\Delta x_{per}=\Delta x_{max}$$

Thus, for distance measurements performed with ultrasonic sensors the absolute error will take the form:

(5) $$\Delta d=\left|d_{m}-d_{r}\right|$$

where $d_{m}$ is the value of the distance obtained by measurement and $d_{r}$ is the real value of the distance. The relative error will be:

(6) $$\varepsilon =\frac{\Delta d}{d_{r}}\times 100\;$$

where Δ*d* is the absolute error and $d_{r}$ is the real value of the distance.

The maximum permissible error will be noted as follows:

(7) $$\Delta d_{per}=\Delta d_{max}$$

- Determining the distance to obstacles of different sizes

**Figure 16:**
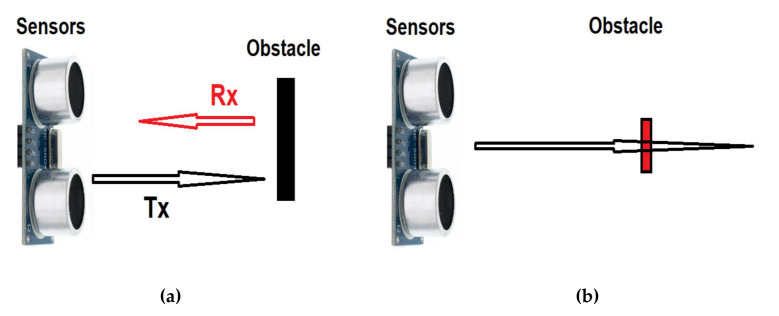
Ultrasonic sensors for detection of small obstacles. Reflection received from targets larger than the wavelength (**a**), and error of detection for smaller objects than the wavelength (**b**).

Another problem with sensors is the detection of small obstacles. Likewise, ultrasonic sensors do not detect objects of very small dimensions—depending on the frequency of the sounding—(Figure 16b) and if they detect them, the determined distance may have values different from the actual distance from the sensor.

The XL Maxbotix EZ0 ultrasonic sensor was used to determine the distance from obstacles of different sizes, in conditions of atmospheric pressure of 714.5 mmHg and temperature of 22.5 °C (295.65 K). In this case, the speed of sound propagation has the value c_air_ = 345.16 m/s.

The XL Maxbotix EZ0 sensor line provides high accuracy and high resolution ultrasonic proximity detection and ranging in air, in a package less than one cubic inch. This sensor line features 1-mm resolution, target-size compensation for improved accuracy, superior rejection of outside noise sources, internal speed-of-sound temperature compensation. This ultrasonic sensor detects objects from 1-mm to 5-m, senses range to objects from 30-cm to 5-m. Sensor operates at 42 kHz. The obstacles used to test the ultrasonic sensor were mounted on the mobile platform and a linear potentiometer guide subassembly was used for tuning the generated sounding intensity. Flat obstacles made of aluminum with a height of 150 mm and a width of 20 mm, 40 mm and 60 mm respectively were used. The distances at which the obstacle was positioned vary from 100 mm to 1000 mm, from 100 to 100 mm.

**Figure 17:**
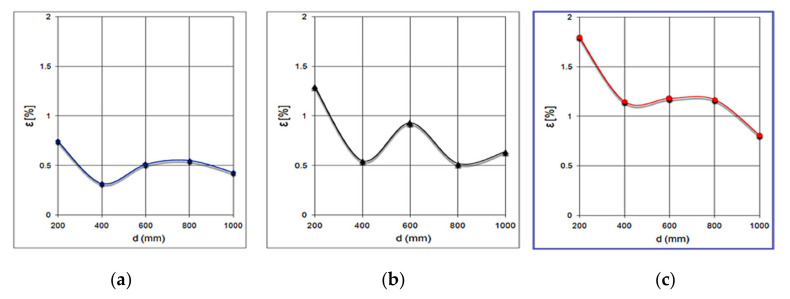
Variations of the relative error for obstacles of different sizes. Error of detection for objects 60 mm wide (**a**), 40mm wide (**b**) and 20 mm (**c**). Error increases inversely with the object width.

Figure 17 shows the variations of the relative error for obstacles of different sizes: (a) the variation of the relative error for obstacles with a width of 60 mm; (b) the variation of the relative error for obstacles with a width of 40 mm; and (c) the variation of the relative error for obstacles with a width of 20 mm.

It is observed that the average value of the relative error increases inversely with the width of the obstacle, which is twice as large at the obstacle with a width of 20 mm compared to that with a width of 60 mm.

- Determining the distance from obstacles of different shapes

The detection of the railway equipment in subway lines may involve objects with different sizes and shapes, with different textures. As in the cases presented above, one another problem with the ultrasonic sensing occurs in the detection of obstacles having different shapes. The measured distance between the sensor and the obstacle may experience different values, depending on the shape of the obstacle.

**Figure 18:**
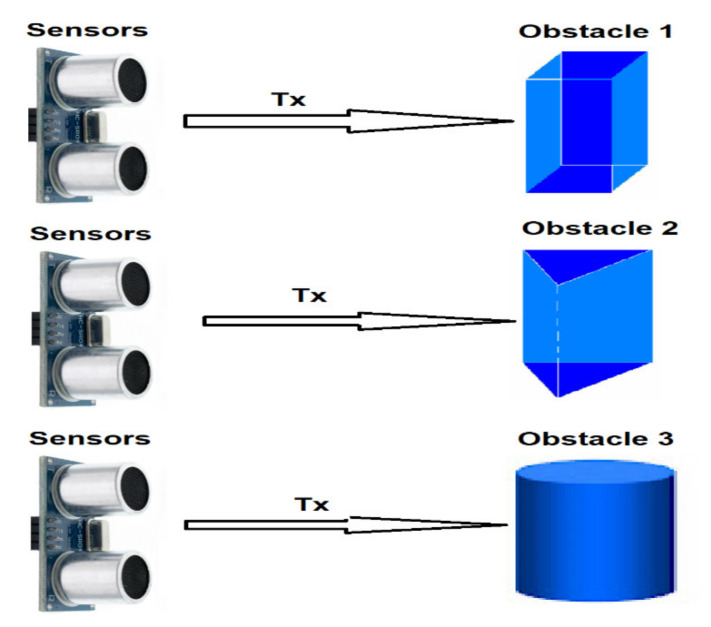
Obstacles of various forms.

To observe the influence of the shape of the obstacle, distance measurements were performed between the ultrasonic sensor XL Maxbotix EZ0 and three obstacles with different cross sections having the dimensions shown in Figure 18. They were positioned in turn at the same distances between 0.2 m and 2 m from the ultrasonic sensor.

To simplify the expression, the obstacle with rectangular section was named Obstacle 1, one with triangular section was labeled Obstacle 2 and the cylindrical obstacle was called Obstacle 3.

**Figure 19:**
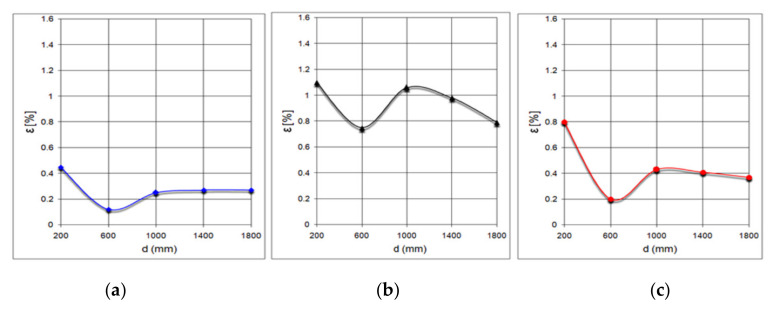
Measuring the distance to obstacles of different shapes. (**a**): variation error for rectangular objects, (**b**): variation error for triangular prism, (**c**): variation error for cylindrical object.

Figure 19 shows the measuring the distance to such obstacles of different shapes: Figure 19a the variation of the relative error for Obstacle 1; Figure 19b the variation of the relative error for Obstacle 2; Figure 19c the variation of the relative error for Obstacle 3.

Measurements with the XL Maxbotix EZ0 ultrasonic sensor were performed at atmospheric pressure of 711.4 mmHg and temperature of 20.6 °C (293.75 K). In this case, the speed of sound propagation has the value c_air_ = 344 m/s.

It can be noted that the average value of the relative error is the highest in the case of Obstacle 2. In addition, the values of the measured distance are the highest in the case of Obstacle 2, which is detected at a greater distance than the actual distance. In the case of cylindrical Obstacle 3, the measured distance values do not differ much from the values obtained for Obstacle 1.

- Determining the distance to obstacles from materials with different textures

In addition to the size and shape of the obstacles, the detection of the ultrasonic sensor can also be influenced by the texture of the material from which the obstacle is made.

To observe the influence of the texture of the material from which the obstacle is made, distance measurements were performed between the XL Maxbotix EZ0 ultrasonic sensor and six obstacles made of different sample materials. These were positioned in turn at the same distances between 50 mm and 250 mm from the ultrasonic sensor. The materials of which the obstacles used for the determinations are made were:

1. Stainless steel;
2. Aluminum;
3. Copper;
4. Wood;
5. Rubber;
6. Plastic.

The field measurements with the XL Maxbotix EZ0 ultrasonic sensor were performed under atmospheric pressure of 705.6 mmHg and temperature of 21.3 °C (294.45 K). In this case, the speed of sound propagation has the value c_air_ = 344.43 m/s.

**Figure 20:**
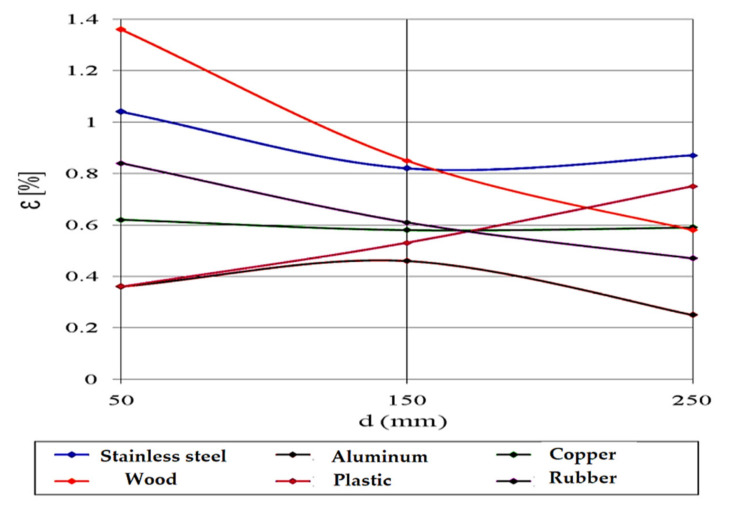
Relative error variation for different materials.

Figure 20 shows the relative error variation for different materials.

When measuring the distance from obstacles made of metals, it can be observed that the values are higher than the actual distance for steel and copper (shiny surface metals) and smaller for aluminum (matt surface metal).

When measuring the distance from obstacles made of non-metals, it can be noticed that the measured values are higher than the actual distance for plastic (glossy surface material) and smaller for wood and rubber (matt surface materials).

- Determining the distance with a sensor mounted on a rotating platform (radar)

To simulate a rotary sensor, measurements were performed with the ultrasonic sensor XL Maxbotix EZ0 located on a mobile platform, which has the possibility to rotate by a maximum of 90 degrees from a fixed point. The experiment was run for 12 s for each obstacle and the sampling rate was 10 samples per second. The first set of measurements was performed without obstacles, determining the dimensions of the workspace.

To simplify the expression, the obstacle with section a rectangle was called Obstacle 1, the one with section a rectangular triangle was called Obstacle 2 and the cylindrical obstacle was called Obstacle 3 (see Figure 18). The obstacles were positioned at the same distance from the ultrasonic sensor.

**Figure 21:**
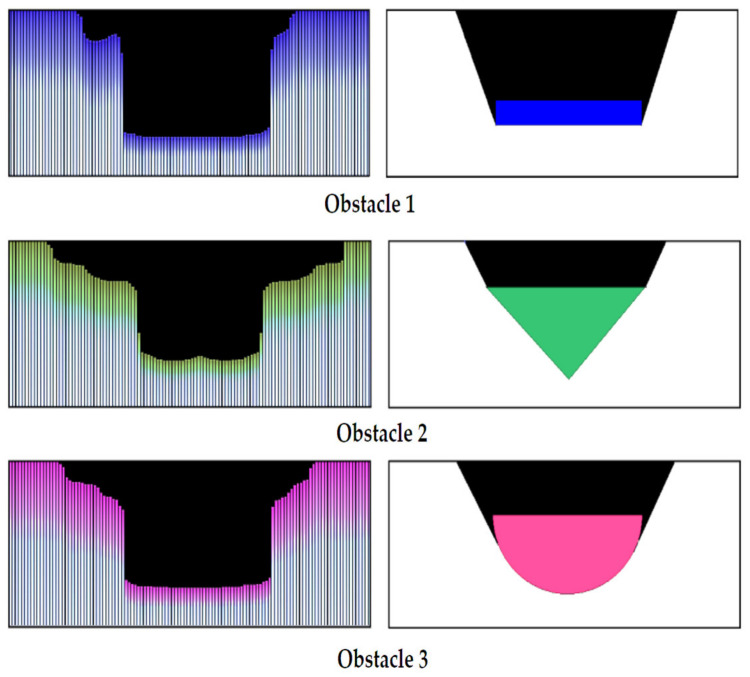
The difference between the real shapes (**right**) and those determined by the radar (**left**).

Following the measurements, the shape of the workspace was determined with each obstacle, as perceived by the ultrasonic sensor. Figure 21 shows the differences in perception of the ultrasonic sensor.

By analysing the three diagrams in Figure 21, one can observe that larger differences in distance measurement occur in case of Obstacle 2 with the section of a right triangle (Prediction 1—blue signal for Obstacle 2). Following experiments with ultrasonic sensors Maxbotix EZ0, it can be said that the accuracy of determining the distance between the sensor and an object is influenced by an object’s size, texture and shape, but also atmospheric parameters, especially temperature and air pressure [53]. However, at the ground level the latter influences are minimal, and the errors determined by the measurements are not large, we recommend the use of ultrasonic sensors for this type of mobile carts.

**Figure 22:**
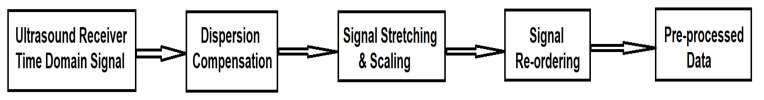
Pre-processing steps of the acquires ultrasound signals.

Figure 22 shows the pre-processing diagram for acquires ultrasound signals.

**Figure 23:**
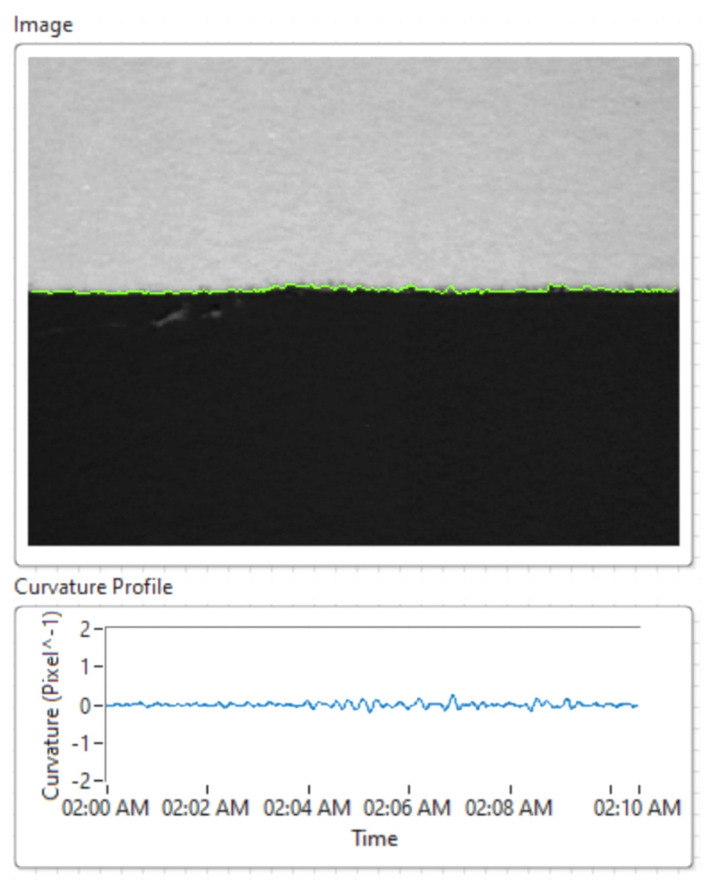
Determining the mechanical deformations of rails based on ultrasonic sensors.

**Figure 24:**
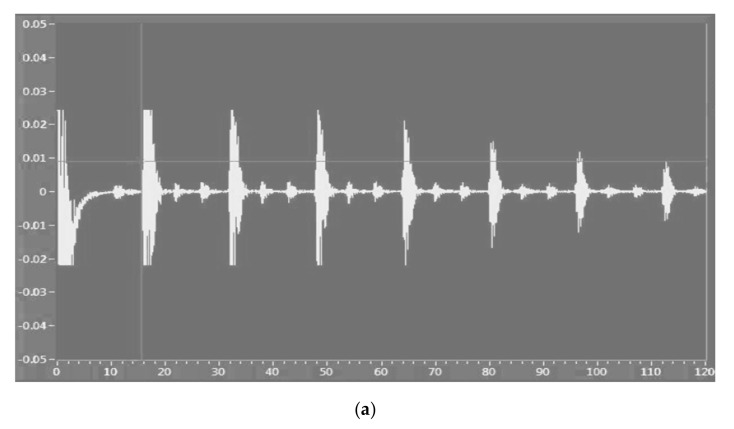

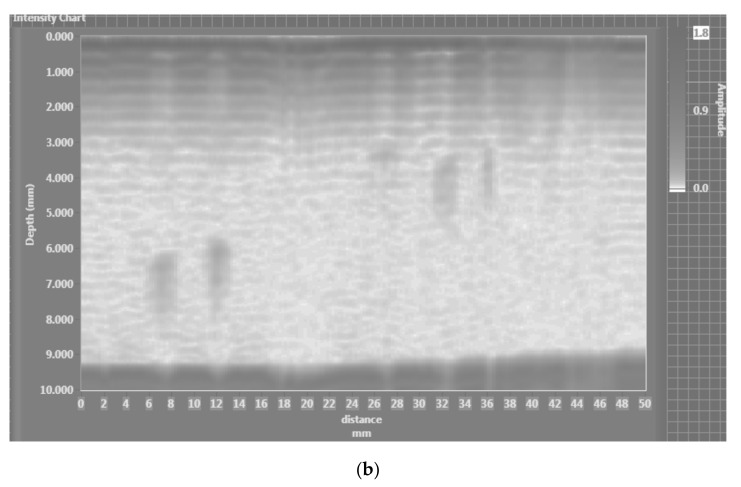
Ultrasonic measurement technique (**a**); data acquisition amplitude modulated scan; (**b**) ultrasonic imaging for the analysis of rail fracturing inside the subway line.

Based on the laboratory tests presented above, we simultaneously analyzed the two subway tracks using an area of ultrasonic sensors based on the analysis of received signals and the representation of ultrasound imaging, to determine the rail profile and the possibilty of identifying mechanical defects inside the rails. The results are shown in Figure 23 and Figure 24.

To construct the PCA T^2^Q model, the input matrix was divided into a training set (for developing the model), and a test set in an odd-even manner. Ultrasonic imaging was employed for detecting subway rail surface quality and/or fracturing.

In Subchapter 3.1 we described the implementation of the machine learning model with the PCA T^2^Q algorithm. During the development and laboratory tests we simulated the model with four ultrasonic sensors that work independently, thus using four components for the PCA T^2^Q algorithm. According to the tests performed to detect subway rail defects, we performed two configurations of ultrasound sensors: the four sensors worked independently and the analysis was performed on PCA 4 components or the grouping of the four sensors in groups of two sensors and PCA analysis was performed with two components. The best results were obtained by grouping the ultrasound sensors in pairs of two sensors each.

**Figure 25:**
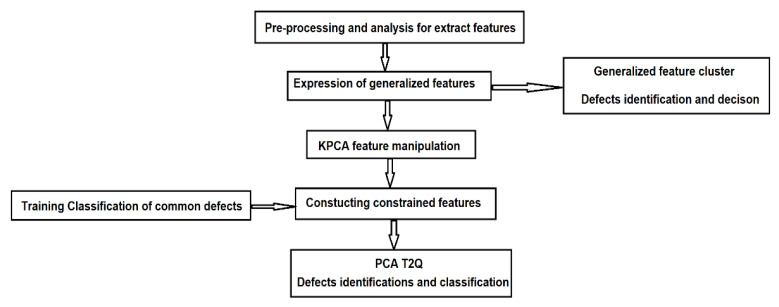
The flow chart of the proposed algorithm.

Figure 25 shows the diagram of the proposed algorithm.

**Figure 26:**
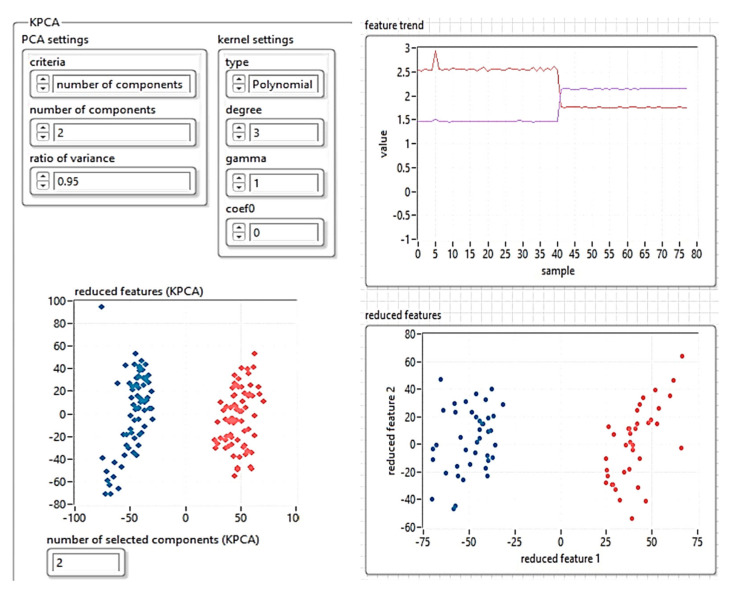
Feature manipulation reducing the database size using the KPCA algorithm.

**Figure 27:**
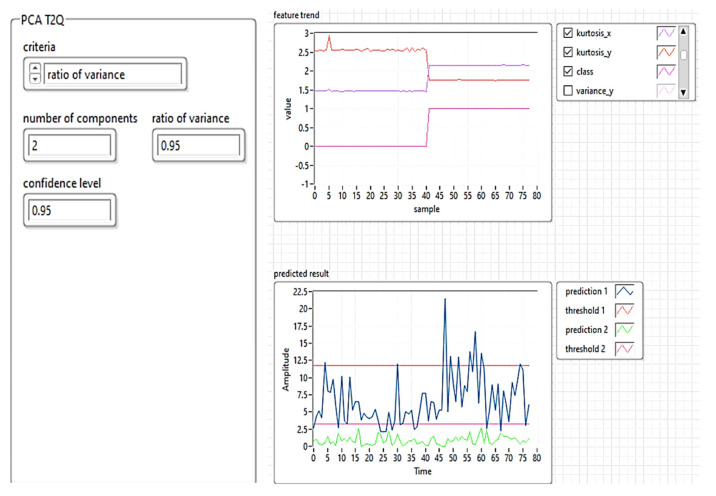
Estimation of rails anomaly detection and predicted result—machine learning modeling using PCA with T^2^Q and ultrasonic sensors.

The algorithm proposed in this research was used to validate the analysis. The results are presented in Figure 26 and Figure 27. Figure 26 shows the feature manipulation reducing the database size using the KPCA algorithm results obtained for the shape of the workspace with each obstacle (see Figure 21) using the ultrasonic sensor.

Figure 27 shows the results obtained for the analysis of defects in the running tracks of the subway using air ultrasonic sensors and the machine learning algorithm with PCA T^2^Q. The prediction results were obtained by correlating the data obtained from ultrasound imaging and determining the running line profile.

## 5. Discussion

To construct the PCA T^2^Q model, the input matrix was divided into a training set (for developing the model), and a test set in an odd-even manner, for enabling the possibility to carry out ultrasonic investigations of metal parts with different geometries and sizes. As a result of the measurements obtained with this system, in repeated experiments, it can be stated that the device met the conditions for which it was designed.

Based on the above considerations, during the laboratory tests, we detected a specific influence of the target edges on the accuracy of the analysis of mechanical deformations at the surface of steel, especially at sound speed measurements. Therefore, the hypothesis is that the margin effect influences the measurements of sound speed, and new experiments must be performed to find solutions that avoid or reduce the effect of edges’ sound reflections.

Although it is necessary to consider the beam propagation when performing an ultrasonic inspection, it is important to note that in the remote field, the maximum sound pressure is always along the sound axis (center line) of the transducer. Therefore, the strongest reflections are likely to come from the front area of the transducer.

The scientific work carried out under the article allowed the analysis of a vast and complex field, the ultrasound investigation, from an applicative-technological perspective. From this perspective, the theoretical research has been further continued experimentally in the field and the results allowed the formulation of the following hypotheses:

- Ultrasonic investigations allow the evaluation of the amplitude in volume, in metal components, with an acceptable accuracy. To obtain the stress values, it is necessary to consider the elastic constants of the material. Further investigations and field tests are necessary in this direction, to also determine the feasibility of using high-frequency ultrasound imaging for subway practical applications.
- Some manufacturing and/or operating and stressing processes (casting, plastic deformation, welding, machining) might introduce into the material of the parts residual stresses that substantially change their operating performance. These are difficult to detect with classic setup of ultrasonic sensors, a deeper investigation is necessary, probably using closer ultrasonic sensing and higher frequencies.
- The great advantages of ultrasonic techniques are the fast data collection, portability of instrumentation, radiation-free control, the possibility of measuring the geometric location of points or continuous time and the low costs. However, to obtain quantitative values of signal amplitude, ultrasonic techniques require the evaluation of elastic properties. Therefore, it is important to precisely know the mission of sound amplitude state analysis.
- Ultrasonic techniques allow the estimation of surface and volume stresses of the parts under investigation.
- The ultrasonic investigation method can be applied to metallic and non-metallic materials, capable of propagating ultrasonic waves with frequencies up to 20 MHz.
- In the analysis of internal structure stresses, decreased propagation time can be interpreted as a decrease in tension or an increase in compression stresses, the change in stress can be approximated using the relative change in time and the appropriate elastic constants.

The algorithm proposed in this article responds well to the instability and to the upper or lower constraints imposed by the characteristics of the train rails during the analysis of the possible defects.

## 6. Conclusions

The scientific approaches presented in this research allowed the analysis of the complex field of ultrasonic investigation, from an applicative—technological perspective.

To make reliable the detection of geometrical defects in the mechanical structure of rails, in this article we proposed a method based on the feature manipulation algorithm with KPCA, and a machine learning structure based on the PCA T^2^Q algorithm to determine generalization characteristics. The main conclusions are as follows:

(1) The proposed method avoids the instability, upper or lower constraints caused by the characteristics of the rails during the processing of anomaly detection. The experimental results showed that the proposed method can work better than similar methods presented, with an accuracy of 98.65% and an average detection time of 0.15 s. However, for better results, the speed of the automated cart should be limited and sampling intervals shortened (for 100 mm sampling intervals, a speed of 0.36 km/h is recommended);
(2) The PCA T^2^Q method is used to group the generalized characteristics constrained after size reduction using KPCA. In addition, the kurtosis index, and the accuracy of the PCA T^2^Q algorithm are used to evaluate the results, and the detection results are obtained with an accuracy of over 95%;
(3) The experimental results show that the proposed method has a higher detection accuracy for rails defects (fracturing, mechanical deformation, and intensive erosion due to rust) and present better application perspectives than the methods reported in the literature.

Future field experiments are still necessary for the development of the integrated solution, which will focus on integrating data from ultrasonic and acoustic sensors with video cameras to detect defects outside and inside the rails using deep learning post-processing. We also seek to investigate the possibility of using closer, higher-frequency ultrasonic sensors for analyzing the rails internal integrity.

## Figures and Tables

**Table 1:** Measurements on the subway rail (ambient temperature: 24 °C).

Sample Interval [mm]	Measured Distance [mm] RP Lidar A1	Measured Distance [mm] XL-Maxsonar EZ *	Measured Distance [mm] BOSCH Professional GLM 50C
100	1403.1	1340	1391
200	1401.0	1350	1390
300	1400.0	1360	1390
400	1400.2	1350	1394
500	1395.8	1350	1394
600	1401.1	1340	1394
700	1403.2	1340	1392
800	1404.1	1340	1394
900	1401.4	1400	1393
1000	1402.8	1350	1393
1100	1403.1	1340	1392
1200	1397.6	1400	1390
1300	1398.3	1350	1390
1400	1400.1	1340	1390
1500	1404.6	1340	1391
1600	1403.6	1350	1390
1700	1402.8	1350	1393
1800	1398.7	1360	1393
1900	1402.3	1340	1390
2000	1400.0	1340	1394
2100	1398.1	1350	1391
2200	1401.2	1350	1391
2300	1399.0	1348	1394
2400	1405.9	1360	1394
2500	1406.3	1340	1393
2600	1404.1	1350	1394
2700	1400.2	1340	1391
2800	1399.1	1350	1392
2900	1401.0	1350	1392
3000	1406.0	1360	1394

## Data Availability

The data used were obtained in the laboratory of Intelligent Transport Systems within the Faculty of Transport, Polytechnic University of Bucharest. The real-time tests were performed in a real subway network in Romania. The data obtained are not public.
